# Bedside Diode Laser Photocoagulation Under Remifentanil Analgesia for Retinopathy of Prematurity: Early Structural Outcomes

**DOI:** 10.4274/tjo.04557

**Published:** 2016-10-17

**Authors:** Mehmet Ali Şekeroğlu, Emre Hekimoğlu, Beyza Özcan, Ahmet Yağmur Baş, Nihal Demirel, Jale Karakaya

**Affiliations:** 1 Ulucanlar Eye Training and Research Hospital, Ankara, Turkey; 2 Zübeyde Hanım Maternity and Research Hospital, Ophthalmology Clinic, Ankara, Turkey; 3 Zübeyde Hanım Maternity and Research Hospital, Neonatology Clinic, Ankara, Turkey; 4 Hacettepe University Faculty of Medicine, Department of Biostatistics, Ankara, Turkey

**Keywords:** laser photocoagulation, remifentanil, retinopathy of prematurity

## Abstract

**Objectives:**

To evaluate one-year structural outcomes of bedside diode laser photocoagulation with remifentanil analgesia for retinopathy of prematurity (ROP) and discuss clinical and demographic characteristics of infants and other possible risk factors that may affect the outcome.

**Materials and Methods:**

The medical records of premature infants who were treated with bedside transpupillary diode laser photocoagulation under remifentanil analgesia for ROP were evaluated for clinical and demographic characteristics, accompanying systemic risk factors, laser parameters, complications of treatment, retreatment rate and one-year structural outcomes.

**Results:**

One-hundred and ninety-five eyes of 99 infants (59 males, 40 females) were recruited for the study. The mean gestational age and birth weight were 27.4±2.3 weeks (23-34) and 1003.3±297.8 g (570-2250), respectively. Laser therapy was performed for high-risk prethreshold ROP in 66.2% of eyes, aggressive posterior ROP (APROP) in 15.4% and threshold ROP in 18.4%. The mean number of laser spots was 1510.4±842.1 per laser session. No adverse effects of laser photocoagulation were observed except small lens opacities in two eyes and corneal opacity in one eye. Retreatment was needed in only three eyes, and vitreoretinal surgery was needed in six eyes of six patients despite laser treatment. Anatomic outcome was favorable in 189 eyes (96.9%) at the end of a 1-year follow-up. Presence of dilated and tortuous iris vessels (p=0.002) and tunica vasculosa lentis (p=0.009) along with type of ROP (APROP and stage 4a ROP at initial presentation) (p=0.001) were associated with poor anatomical outcome.

**Conclusion:**

Accurate and timely bedside transpupillary diode-laser photocoagulation under remifentanil analgesia is an effective and safe treatment modality for ROP, and may prevent vision-threatening retinal detachment and reduce the need for vitreoretinal surgery.

## INTRODUCTION

Approximately 25,000 low birth weight babies are born each year in Turkey, and about 1,000 of these babies are at high risk of blindness.^1,2^ Retinopathy of prematurity (ROP) is a proliferative vitreoretinopathy arising from the avascular retina in premature neonates. Low gestational age and low birth weight are known to be the main risk factors of ROP.^3^ Previous studies have reported the effect of various factors in the development of ROP, including prolonged mechanical ventilation, excessive oxygen use, bronchopulmonary dysplasia, surfactant treatment, apnea, anemia, blood transfusion, sepsis, hyperbilirubinemia, intraventricular hemorrhage, candidemia, maternal preeclampsia, maternal diabetes, multiples pregnancy, and chorioamnionitis.^4,5^ Whether these factors are truly independent risk factors leading to ROP or emerge secondarily to already existing prematurity remains controversial. With the increasing use of assisted conception techniques, multiples pregnancy and premature birth rates are rising. Advances in premature neonatal care have greatly increased survival rates among very low birth weight infants, but the high comorbidity rates in these infants has made ROP a serious health issue, particularly in developing nations. Despite improvements in diagnosis and treatment, ROP continues to be one of the leading causes of childhood blindness in developing countries.^6^ The aim of ROP screening examinations is to prevent blindness through timely detection and appropriate treatment of high-risk patients.^7^

ROP treatment protocols are created based on the multi-center cryotherapy (CRYO)-ROP and Early Treatment for Retinopathy of Prematurity (ETROP) studies.^8,9^ In the CRYO-ROP study, cryoablation of the entire avascular retina was recommended for patients with threshold disease.^8^ It was later thought that treating threshold disease may be too late, and the ETROP study evaluated laser photocoagulation of the avascular retina in patients with high-risk prethreshold disease (zone I, any stage ROP with plus disease; zone I, stage 3 ROP without plus disease; zone II, stage 2 or 3 ROP with plus disease).^9^ The ETROP study reported treatment of prethreshold disease reduced unfavorable functional outcomes from 19.5% to 14.5% and unfavorable structural outcomes from 15.6% to 9.1%. Thus, laser photocoagulation of the entire retinal avascular field at the high-risk prethreshold stage is currently accepted as the safest and most effective approach. Laser photocoagulation is usually performed in operating room conditions under general anesthesia. However, in cases where general anesthesia cannot be used or the associated risks should be avoided, laser photocoagulation can be performed in the neonatal intensive care unit under alternative anesthesia methods.^10,11,12^

In the present study we analyzed the records of premature infants with ROP treated by transpupillary diode laser photocoagulation under remifentanil analgesia in the neonatal intensive care unit in order to present 1-year anatomic outcomes and discuss the probable impact of clinical and demographic characteristics on those outcomes.

## MATERIALS AND METHODS

After receiving approval from the ethics committee, the medical records of 99 premature babies who were diagnosed with ROP and underwent transpupillary 810 nm diode laser photocoagulation under remifentanil in the neonatal intensive care unit between October 2010 and September 2012 were analyzed retrospectively. The study group included both neonates born and followed in our hospital and neonates admitted to the neonatal intensive care unit of our hospital for treatment following ROP diagnosis at other centers. The following data were recorded for all patients: ROP stage before treatment; anterior segment findings such as tunica vasculosa lentis and iris vascular dilation and tortuosity, detected by a portable, handheld biomicroscope (XL-1, Shin-Nippon, Japan); timing of laser treatment and laser settings used; treatment response and retina examination findings at 1 year. Patients with attached retina and without optic disc shrinkage or tractional membranes were categorized as group 1; patients with retinal detachment, optic disc shrinkage and/or tractional membranes were categorized as group 2. Furthermore, the patients’ records were analyzed for the presence of neonatal and maternal risk factors such as gestational age, birth weight, APGAR scores at 1 and 5 minutes, mode of delivery, gender, multiples pregnancy, preeclampsia, maternal diabetes, early membrane rupture, placenta ablatio, blood transfusion, clinical sepsis, respiratory distress syndrome, necrotizing enterocolitis, intracranial hemorrhage, hydrocephaly, and assisted conception techniques.

Prior to treatment, 2.5% phenylephrine (Mydfrin^®^, Alcon, USA) and 0.5% tropicamide (Tropamid^®^, Bilim İlaç, Turkey) eye drops were instilled 3 times at 10 minute intervals. The patients were sedated using 0.1 mg/kg midazolam administered by intravenous bolus by a neonatal specialist and intubated. Intravenous infusion of 0.2 to 0.6 µg/kg/min remifentanil was performed under the supervision of a neonatal specialist while monitoring life signs and level of analgesia. After achieving sufficient pupil dilation and sedoanalgesia, 0.5% proparacaine hydrochloride (Alcaine^®^, Alcon, USA) was instilled immediately before laser treatment as local anesthesia. Panretinal photocoagulation was applied with a 810 nm diode laser (Iridex, Oculight SL, USA) to the entire avascular zone leaving half-spot intervals (200-400 mW power; 0.2-0.3 second exposures). In eyes with stage 4a ROP, the laser was also applied twice to the vascular area posterior to the detachment. The laser photocoagulation procedure was performed while monitoring life parameters in the presence of a neonatal specialist. None of the patients developed any anesthesia-related problems which complicated the laser treatment.

SPSS for Windows version 21.0 (Statistical Package for the Social Sciences Inc., Chicago, IL, USA) software was used for statistical analyses. Student’s t test, Mann-Whitney U test and Wilcoxon test were used in comparison of the variables. Normality of data distribution was determined by Kolmogorov-Smirnov test. Descriptive statistics were expressed as frequency and percent for qualitative data, as mean ± standard deviation for quantitative data with normal distributions and median (minimum-maximum) for non-normal distributions. Results with p values less than 0.05 were accepted as statistically significant.

## RESULTS

The medical records of 99 neonates were retrospectively analyzed. Fifty-nine (59.6%) patients were male and 40 (40.4%) were female. Mean gestational age was 27.4±2.3 (23-34) weeks and mean birth weight was 1,003.3±297.8 (570-2,250) g. Gestational age was 28 weeks or less for 74 patients (74.7%), 29-32 weeks for 20 (20.2%) and over 32 weeks for 5 (5.1%). The mean age was 26.17±1.50 weeks in the 18 neonates who underwent laser therapy for aggressive posterior ROP (APROP) and 27.69±2.40 weeks in the other neonates (p=0.001). Gestational age was 837.2±170.4 g in the neonates with APROP and 1040.2±308.1 g in the others (p=0.008).

A total of 195 eyes of 99 neonates were laser treated (bilaterally in 96 patients, unilaterally in three patients) at mean postmenstrual 36.9±2.3 (33-43) weeks, at a mean chronological age of 66.9±18.9 (20-110) days; a mean of 1,510.4±842.1 (381-5258) laser pulses were applied at half-spot intervals to the entire avascular retina. Laser therapy was applied in 129 eyes (66.2%) at the high-risk prethreshold disease stage as per the ETROP study, 30 eyes (15.4%) with threshold disease as per the CRYO-ROP study, and in 36 eyes (18.4%) with APROP. We observed that there were differences in treatment indications and that in some patients, treatment delay was a result of delayed referral to our clinic from other medical centers. In the 6 eyes that presented with stage 4a disease limited to 2-3 clock hours, an additional 2 laser applications were applied to the ridge tissue behind the vascular retina. Mean regression time of ROP after laser therapy was 4.5±1.8 (3-10) weeks. A second session of laser treatment was done a mean 9.0±5.3 days (5-15 days) later for 3 patients due to insufficient treatment response and/or untreated areas. Anterior segment complications occured in 3 eyes (small lens opacity in 2 eyes, corneal opacity in 1 eye) after laser therapy.

Retinal attachment was observed in 189 (96.9%) of the laser-treated eyes for the 1-year follow-up period (group 1). Six eyes (3.1%) were referred to another center for vitreoretinal surgery due to retinal detachment (group 2; 2 eyes with new detachment following APROP, 3 eyes with stage 4a disease detachment not resolved by laser therapy). Patients with unfavorable anatomic outcomes had lower average birth weights and gestational ages, higher chronologic and postmenstrual ages at time of laser application, and lower APGAR scores at 1 and 5 minutes, but these differences were statistically nonsignificant (Table 1). APROP, delayed laser treatment (stage 4a), tunica vasculosa lentis prior to treatment, and iris vascular dilation/tortuosity emerged as significant risk factors for unfavorable anatomic outcomes (p=0.001, p=0.001, p=0.009, p=0.002, respectively). APROP was observed in all 6 neonates with abnormal anterior segment findings like tunica vasculosa lentis and iris vascular dilation and tortuosity; of these, 3 eyes of 3 patients required vitreoretinal surgery due to new retinal detachment. The relationships between other neonatal and maternal risk factors and anatomic outcomes at 1-year follow-up are shown in detail in Tables 2 and 3.

## DISCUSSION

The method currently accepted as safest and most effective for the management of ROP is laser photocoagulation of the entire retinal avascular field at the high-risk prethreshold stage. The procedure is traditionally performed in an operating room under general anesthesia and yields favorable anatomic outcomes at high rates when applied appropriately and in a timely manner. In the present study, we found that anatomic success at 1 year was achieved in 96.9% of eyes that underwent laser photocoagulation of the avascular field performed in the neonatal intensive care unit under sedoanalgesia with remifentanil.

The growing number of neonatal intensive care facilities have led to higher rates of various complications associated with prematurity, especially ROP.^6^ Low birth weight and gestational age are the main risk factors for ROP.^5^ For this reason, international screening guidelines generally recommend screening neonates born at a gestational age less than 32 weeks or birth weight under 1,500 g.^13^ However, particularly in developing nations, older neonates have also been reported to develop ROP which may require treatment due to less than ideal neonatal intensive care conditions.^14,15,16,17^ In our study, 5 neonates (3.9%) with gestational ages over 32 weeks required laser treatment, and 1 neonate born at 33 weeks developed retinal detachment despite laser treatment. Therefore, it is important that screening programs be designed according to local conditions and that screening also include neonates at gestational ages over 32 weeks who have additional risk factors or are indicated for screening by a neonatal specialist.

The conventional treatment for ROP is laser photocoagulation of the avascular retinal fields performed under general anesthesia in operating room conditions. In some cases, however, general anesthesia cannot be used or the associated risks should be avoided, such as in the absence of an anesthesiologist experienced in neonatal anesthesia or in patients with concomitant systemic conditions. In such cases, laser photocoagulation can be applied in the neonatal intensive care unit using alternative means of anesthesia.^10,11,12^ Especially in developing countries, ROP screening examinations are usually performed in obstetric and gynecologic hospitals, which may not always have an anesthesiologist experienced in neonatal anesthesia. When general anesthesia is not applicable, patients are referred to other centers which are able to administer general anesthesia for urgent treatment of ROP. However, disease progression during the time required to transfer the patient decreases the chance of favorable treatment outcomes. Three of the infants that required vitreoretinal surgery due to retinal detachment were not able to receive treatment before the disease reached stage 4a. This demonstrates how serious the consequences of delaying treatment during the process of referring patients to other centers can be. Furthermore, 1 of these 3 infants was born at a gestational age of 33 weeks and a birth weight of 1,690 g, illustrating that delayed treatment can result in irreparable damage even in infants that are not considered high-risk. Even when institutional conditions permit the use of general anesthesia, an infant’s general condition may deteriorate while being transferred from the neonatal intensive care unit to the operating room, and extubation takes time following general anesthesia. Laser therapy for ROP is generally performed between postnatal weeks 6 and 8; for a premature infant who has been very recently extubated, postsurgical extubation will also take time. As a result, laser therapy is increasingly performed under topical anesthesia and sedation as an alternative to general anesthesia. For all these reasons, we also conduct laser photocoagulation therapy in the neonatal intensive care unit under the guidance of a neonatal specialist, without giving general anesthesia. Although general anesthesia may be ideal in terms of keeping the infant motionless and thus facilitating laser application, performing laser therapy under remifentanil analgesia provides anatomic success without causing any lasting systemic complications.

Remifentanil is an ultra short-acting synthetic opoid analgesic drug. Its rapid plasma clearance, rapid effect onset and cessation as soon as the infusion is stopped make it suitable for use in premature infants.^11,12^ Sammartino et al.^11^ reported that performing laser therapy under remifentanil infusion provided optimal surgery stress control with no side effects and found that premature infants were able to return quickly to preoperative respiratory function. A study analyzing premature infants’ pain scales showed that the infants did not experience any severe pain during laser application under remifentanil anagesia.^12^ The authors of that study reported transient hypotension and bradycardia in 2 of the 64 infants treated for ROP with laser therapy under remifentanil infusion; the remaining infants experienced no side effects associated with anesthesia. They concluded that this procedure is effective, reliable and practical in hospitals where access to pediatric anesthesiologists is limited. Our retrospective chart review of infants who underwent laser therapy under remifentanil analgesia did not include analysis of the effectiveness or side effects of this anesthesia method. However, there were no incidences of anesthesia-related difficulties with laser application.

In the present study, we found that anatomic success at 1 year was achieved in 96.9% of eyes that underwent laser photocoagulation of the avascular field performed in the neonatal intensive care unit under sedoanalgesia with remifentanil. However, due to possible refractive errors, anisometropia, strabismus, late-stage retinal tears and detachment as well as cortical causes, functional success rates may not be as high as anatomic success rates. In the current study we present only anatomic outcomes because other factors which may influence functional success, such as refractive errors and strabismus, were not analyzed. Another limitation of the study is the small number of patients requiring vitreoretinal surgery due to retinal detachment. A study including a larger patient group may reveal different risk factors statistically associated with retinal detachment.

APROP has poor prognosis and is more difficult to treat than classic ROP. The extreme vascular activity of this disease results in a very high rate of unfavorable anatomic and functional outcomes.^18^ Therefore, some researchers recommend early vitrectomy.^19,20^ However, early vitrectomy is not currently a widely accepted practice. It was reported that off-label use of intravitreal bevacizumab injections with or before laser therapy positively influences prognosis and resulted in better outcomes than laser photocoagulation alone in zone I disease.^21^ Regardless, as the possible side effects and long-term safety of intravitreal therapies in premature infants still in early development are not known, they should be used with caution.

## CONCLUSION

In conclusion, we achieved a high rate of anatomic success at postoperative 1 year by performing laser photocoagulation therapy in the neonatal intensive care unit under sedoanalgesia with midazolam and remifentanil. The main risk factors for unfavorable anatomic outcomes were APROP, delayed laser therapy (stage 4a) and abnormal anterior segment findings such as tunica vasculosa lentis or increased iris vascular dilation and tortuosity prior to treatment. Despite satisfactory anatomic outcomes at 1 year, desired functional outcomes may not be achievable due to various reasons including refractive errors, anisometropia, strabismus, cortical causes, and late-stage retinal tears and detachment.

### Ethics

Informed Consent: Consent form was filled out by all participants.

Peer-review: Externally peer-reviewed.

## Figures and Tables

**Table 1:**
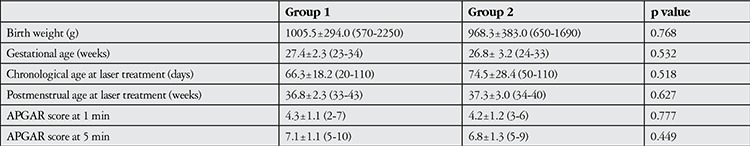
Comparison of selected clinical characteristics of patients in group 1 and group 2

**Table 2:**
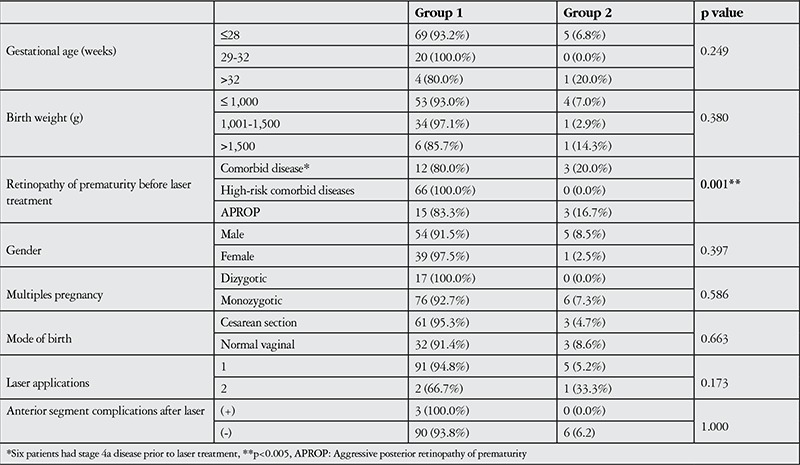
Comparison of selected clinical and demographic characteristics of patients in group 1 and group 2

**Table 3:**
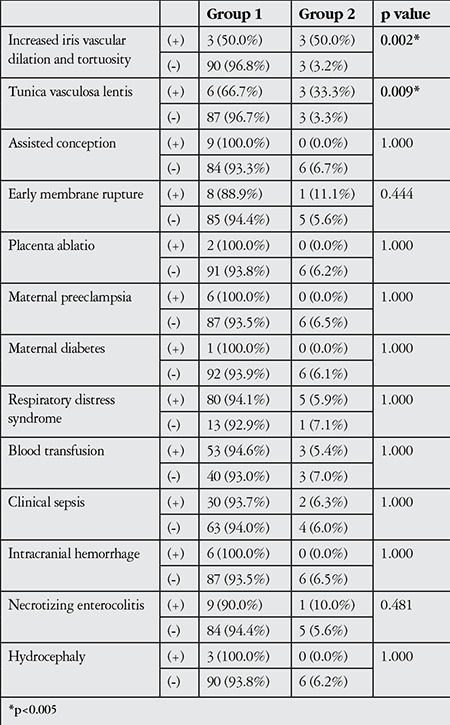
Comparison of neonatal and maternal risk factors of patients in group 1 and group 2
